# Unraveling the roles of WRN and DNA-PKcs at telomeres

**DOI:** 10.18632/aging.100150

**Published:** 2010-05-27

**Authors:** Tara L. Beattie, Susan P. Lees-Miller

**Affiliations:** Departments of Biochemistry and Molecular Biology and Oncology and The Southern Alberta Cancer Research Institute, University of Calgary, Calgary, AB, Canada T2N 4N1

In eukaryotic cells, the ends of linear chromosomes are maintained by nucleoprotein complexes called telomeres, which are composed of long stretches of repetitive DNA sequences bound by specific telomere binding proteins. The repetitive DNA sequence (repeats of TTAGGG in humans) is composed of double stranded DNA and, at its extreme end, a single-stranded G-rich 3' overhang, referred to as the G-tail. The telomere folds back on itself to form a lariat-shaped loop called the T (for telomere) loop, and the G-tail invades the double stranded DNA of the T-loop to form a D (for displacement) loop. This entire structure is stabilized by interaction with proteins of the shelterin complex, namely TRF1, TRF2, TIN2, RAP1, TPP1 and POT1 [1,2]. The T-loop structure is thought to protect the ends of linear chromosomes from being recognized by the cell as DNA double strand breaks (DSBs) [3]. Paradoxically, numerous proteins known to be involved in the detection and repair of DSBs are also found at telomeres. One such protein is the DNA-dependent protein kinase catalytic subunit, DNA-PKcs, which plays a critical role in the repair of DSBs by non-homologous end-joining in mammalian cells [4,5]. Another is the Werner Syndrome protein, WRN, a multifunctional protein with 3'-5' DNA helicase and 3'-5' exonuclease activities [6].  Both DNA-PKcs and WRN have functions not only in DNA repair and maintenance of genomic stability, but also in the maintenance of telomere integrity. Loss of DNA-PKcs results in telomere uncapping and chromosomal fusions [7], whereas loss of WRN results in telomere shortening and is associated with a premature aging phenotype [6]. One of the current challenges in telomere biology is to understand how these proteins and protein-DNA complexes regulate telomere length, maintain telomere integrity, and prevent the initiation of a DNA damage response and cellular senescence.

In this issue of Aging, Kusumoto-Matsuo and colleagues [8] report that DNA-PKcs stimulates the helicase activity of WRN towards model D loop structures in vitro. DNA-PKcs did not affect the exonuclease activity of WRN or the unwinding activity of the related RecQ helicase, BLM, suggesting that this effect is specific for the WRN helicase activity.  Significantly, WRN and DNA-PKcs were shown to cooperate to protect the 3' single stranded ends of telomeres in vivo by preventing G-tail shortening. These findings have important implications for how DNA-PKcs and WRN cooperate to resolve these complex telomeric DNA structures. Moreover, since shortening of the G-overhang contributes to onset of cellular senescence [9], these findings suggest that the WRN/DNA-PKcs interaction might contribute to preventing cellular senescence.

This study is the first to report a functional interaction between WRN and DNA-PKcs at telomeres and provides a stepping-stone to further elucidate the critical roles that these proteins play in telomere integrity and cellular senescence. It also raises some interesting questions. For example, it will be interesting to determine whether other proteins present at telomeres such as TRF2, which has recently been shown to regulate the activity of WRN [10], affects the regulation of WRN by DNA-PKcs. Equally interesting will be to determine the molecular mechanism by which DNA-PKcs stimulates the helicase activity of WRN and whether the DNA-PKcs binding partner, Ku, plays a role in this process.   It will also be interesting to determine whether WRN-mediated unwinding promotes telomerase accessibility to the telomere for example during S-phase. Interestingly, the authors have also shown that DNA-PKcs stimulates the activity of WRN towards D-loops composed of non-telomeric DNA sequences, suggesting that these findings may extend to D-loop structures formed during other nuclear processes such as homologous recombination. In conclusion, these findings bring us one step closer to unraveling the complex interplay between DNA damage response proteins and telomeres and to understanding how these complex and dynamic structures contribute to the maintenance of genomic stability and the prevention of premature aging.
